# A comparison of the effects of *Portulaca oleracea* seeds hydro-alcoholic extract and Vitamin C on biochemical, hemodynamic and functional parameters in cardiac tissue of rats with subclinical hyperthyroidism

**Published:** 2018

**Authors:** Hadi Khodadadi, Roghayeh Pakdel, Majid Khazaei, Said Niazmand, Kowsar Bavarsad, Mousa AL-Reza Hadjzadeh

**Affiliations:** 1 *Neurogenic Inflammation Research Centre, Mashhad University of Medical Sciences, Mashhad, Iran*; 2 *Department of Physiology, Faculty of Medicine, Mashhad University of Medical Sciences, Mashhad, Iran*; 3 *Division of Neurocognitive Sciences, Psychiatry and Behavioral Sciences Research Center, Mashhad University of Medical Sciences, Mashhad, Iran*; # * The role of authors is equal*

**Keywords:** Portulaca oleracea seeds, Vitamin C, Heart, Subclinical hyperthyroidism, Rat

## Abstract

**Objective:**

The present study was performed to evaluate the effects of hydro-alcoholic extract of *Portulaca oleracea* (*P. oleracea*) seeds and Vitamin C on biochemical and hemodynamic parameters in cardiac tissue of rats with subclinical hyperthyroidism.

**Materials and Methods:**

Forty eight male rats were divided into six groups of 8 and treated for 4 weeks. T4 group received daily injection of levothyroxine sodium (20 μg/kg) and control group was given daily injection of saline. T4-Po groups were given T4 plus 100, 200, and 400 mg/kg of *P. oleracea* seeds extract in drinking water daily. T4-Vit C group received T4 plus daily injection of Vitamin C (100 mg/kg). At the end of the experiment, body weight, serum free T4 level, left ventricular developed pressure (LVDP), malondialdehyde (MDA) and total thiol levels were measured.

**Results:**

Free T4 levels were increased in all groups that were treated with T4. Weight gain was decreased in T4 and T4-Po100 groups compared to control group (p<0.001 and p<0.05). However, body weight was increased in T4-Po (200 and 400) and T4-Vit C groups compared to T4 group. LVDP was increased in T4 group compared to control group but, LVDP was decreased in T4-Po and T4-Vit C groups. Malondialdehyde was decreased in T4-Po groups and T4-Vit C group compared to T4 group. Total thiol groups were increased in T4-Po (200 and 400) and T4-Vit C groups compared to T4 group.

**Conclusion:**

The results showed that *P. oleracea* extract has a protective effect on cardiac dysfunction due to subclinical hyperthyroidism induced by levothyroxine sodium in rats.

## Introduction

Cardiovascular effects of thyroid hormones have been known from a long time. Extensive evidences indicate that the cardiovascular system responds to the fluctuations in the levels of circulating thyroid hormones (Fazio et al., 2004 ▶). Cardiac function alters in both hypo- and hyperthyroid states.

Subclinical hyperthyroidism (SHT) is defined as levels of serum thyroid hormones especially free T4 level within the reference range (close to the maximum normal range) with low or no detectable serum TSH concentration (Toft, 2001 ▶). Many patients with subclinical hyperthyroidism are accidentally diagnosed during health screening conducted by family physicians. 

Heart rate, left ventricular mass, and the risk of atrial arrhythmias and fibrillation (AF) are increased in subclinical hyperthyroidism (Fazio et al., 2004 ▶). The rapid and irregular heart beat produced by AF increases the risk of blood clot formation inside the heart. These clots may eventually become dislodged (Bielecka-Dabrowa et al., 2009 ▶). 

Oxidative stress is defined as excess formation of molecules such as reactive oxygen species (ROS) in the body. Excess thyroid hormones induces hyper metabolic state and this condition is associated with tissue oxidative damage. Under hyperthyroidism conditions, tissues exhibit an increased ROS production (Venditti and Di Meo, 2006 ▶), spatially a significant increase in lipid peroxidation in heart tissue(Gredilla et al., 2001 ▶). 

Vitamin C, also known as L-ascorbic acid, is a water-soluble vitamin that is naturally present in many fruits and vegetables. Humans, unlike most animals, are unable to synthesize vitamin C; so, it is regarded as an essential dietary component (dos Reis-Lunardelli et al., 2007 ▶).

It has been reported that increased intake of antioxidants such as vitamin C, ameliorate cardiovascular diseases (Zhang et al., 2014 ▶) and can reduce oxidative damage in heart tissue. In addition to its antioxidant functions, vitamin C plays an important role in the immune system. This vitamin has been shown to regenerate other antioxidants within the body, including alpha-tocopherol (vitamin E) (Jacob and Sotoudeh 2002 ▶).

*Portulaca oleracea* is a summer annual vegetable (Portulacaceae family) which is grown in many countries (Sultana and Rahman 2013 ▶). This half-hardy low growing plant has slightly succulent leaves and stems that are consumed raw or cooked. There are green and yellow leaved forms; the green type has thinner leaves, is more vigorous and possibly better flavored (Karimi et al., 2010 ▶).

In folk medicine, it is utilized as an antipyretic, anti-scorbutic, anti histamine, antiseptic, antispasmodic, diuretic and anthelmintic agent (Chan et al., 2000 ▶). The aerial parts of the plant have anti-inflammatory (Kaveh et al., 2017) and pain-relieving effects (Hajzadeh et al., 2004 ▶). Recent pharmacological studies have shown muscle relaxant (Okwuasaba et al., 1987 ▶), anti histamine and β-adrenergic stimulatory (Boskabady et al., 2016 ▶; Hashemzehi et al., 2016 ▶), locomotor activity reducing, anti-convulsant, analgesic, and anti-inflammatory effects as well as antioxidant properties of this plant (Chan et al., 2000 ▶). *P. oleracea* seeds have been used in hematuria, gonorrhea, dysuria, strangury and diseases of kidney, bladder and lungs (Sultana and Rahman, 2013 ▶). The decoction from seeds powder are used as a vermifuge and the decoction is useful in gonorrhea (Dweck, 2013 ▶). The seeds also possess diuretic and anti-dysenteric activities (Sultana and Rahman 2013 ▶)

It was shown that *P. oleracea* is a rich source of omega-3 fatty acids, gallotannins, kaempferol, quercetin, apigenin, glutathione (Yen et al., 2001 ▶), alkaloids vitamins (mainly vitamin A, vitamin C, and some vitamin B and carotenoids), as well as dietary minerals, such as calcium, magnesium, potassium and iron (Yazici et al., 2007 ▶).

To the best of our knowledge, there is no study comparing the ameliorative effects of *P. oleracea* seeds and vitamin C on cardiac parameters in subclinical hyperthyroidism; therefore, this study was carried out to compare the effect of hydroalcoholic extract of *P. oleracea* seeds and vitamin C on biochemical, hemodynamic and functional parameters in cardiac tissue of rats with subclinical hyperthyroidism.

## Materials and Methods

**Animals**

This study was conducted on 48 male Wistar rats (200±20 g) supplied from the animal house of Medical School of Mashhad University of Medical Sciences. The rats were kept under standard conditions of temperature (21±2 ^°^C) and light (12 h dark and 12 h light) and fed with a standard diet and water *ad libitum*. Animal care and handling were performed according to the guidelines set by the Iranian Ministry of Health and Medical Education for laboratory animals. Study protocol was approved by the Ethics Committee of Mashhad University of Medical Sciences. The rats were divided into six groups (n=8) and treated for 28 days as follow: 

Group 1) Control group: The rats received normal saline interaperitoneally (i.p) at a volume similar to that of T4 group.

Group 2) T4 group: Levothyroxine sodium was given (20 µg/ kg /day, i.p) for 28 days.

Groups 3- 5) T4-Po groups (T4-Po100, T4-Po200, and T4-Po400): These rats received levothyroxine sodium (20 µg/kg, i.p) plus *P. oleracea* 100, 200 and 400 mg/kg in drinking water for 28 days.

Group 6) T4-Vit C: Levothyroxine sodium was given (20 µg/ kg /day, i.p) plus vitamin C (100 mg/ kg, i.p) for 28 days.

**Plant extraction**

*P. oleracea* seeds were purchased from Imam Reza pharmacy store and their identity was confirmed in herbarium Institute of Mashhad Ferdowsi University (Herbarium number: 240-1615-12). Soxhlet method was used for extract preparation. Seeds were powdered by a mechanical grinder and 100 g of the powder was mixed with 70% ethanol and put on a Soxhlet extractor.

The resulting extract was condensed under reduced pressure and kept in the refrigerator at 4^°^C until used. The weight of the dried extract was 11 g (i.e. extraction yield: 11% w/w). Finally, the extract was dissolved in distilled water to prepare the proper doses. 

**Measurement of cardiac hemodynamic parameters**

Cardiac hemodynamic parameters were measured at the end of the experiment. Animals were anesthetized by i.p injection of ketamine 90 mg/kg and xylazine 10 mg/kg. Right carotid artery was cannulated with a PE 50 catheter connected to a pressure-transducer connected to an amplifier. Then, the catheter entered the left ventricle of the heart, and the cardiac parameters were recorded for 10 min and saved in Lab chart software. 

Left ventricular developed pressure (LVDP) reflects the difference between systolic and diastolic ventricular pressure (mm Hg). Increased LVDP is an indicator of hyperdynamic heart and calculates according to the following formula:

 LVDP = LVSP (Left ventricular systolic pressure – LVEDP (Left ventricular end diastolic pressure) (Golshahi, 2004 ▶).

**Tissue preparation**

The animals were killed by deep ether anesthesia. The heart was rapidly excised, placed into petri dish containing ice-cold isolation medium, rinsed to become free of blood and weighed; then, it was homogenized by phosphate buffered saline (PBS) solution for biochemical studies.

**Measurement of reactive compounds with thiobarbituric acid (TBARS)**

Lipids are among the most important molecules invaded by free oxygen radicals. The most important product of free radicals due to lipids peroxidation, is malondialdehyde (MDA). To determine lipid peroxidation levels, TBARS was measured in heart tissue. MDA reacts with thiobarbituric acid (TBA), and creates a red complex that has peak absorption at 535 nm.

For measurement of MDA, TBA solution was prepared as follows: 375 mg of TBA was added to 2 ml hydrochloric acid (HCl) and the final mixture was added to 100 ml of tri-chloroacetic acid (TCA) 15% solution. Then, 1 ml of homogeneous tissue mixture (supernatant) was mixed with 2 ml of this solution (TBA-TCA-HCL) and after heating and centrifuging at 1000 rpm, absorption (A) at 535 nm was measured using spectrophotometer and was expressed as: nmol/g tissue. Finally, the TBARS concentration was calculated by using the following formula (Janero 1990 ▶):

TBARS C (M) = A/1.65 × 10^5^

**Measurement of total thiol groups**

For determination of total thiol groups as indicators of protein oxidation, DTNB reagent which reacts with SH groups and produces a yellow complex (anion nitro mercapto benzoate) with a peak absorption at 412 nm. 

To 50 μl of homogeneous sample, 1 ml of Tris-EDTA buffer (10 mM Tris and 1 mM EDTA; pH 8.0) was added and its absorption was measured at 412 nm against the Tris-EDTA buffer (A1). Then, 20 μl of DTNB (10 mM DTNB in methanol) was added to homogeneous sample and after about 15 min, the sample absorption was re-measured (A2). The DTNB absorption of the solution was also read as blank (B) and was expressed as: µmol/g tissue. Finally, the amount of total thiol groups was calculated by using the following formula (Ellman 1959):

Amount of total thiol groups = (A2–A1-B) × (1.07 / 0.05) × 13.6

**Thyroid hormone measurement**

Blood samples were collected in test tubes via arterial catheter, and immediately centrifuged at 3000 rpm for 15 min. The serum was separated and frozen at -80 ^°^C for later analyses. Serum thyroid hormones levels were measured with a chemiluminescence immunoassay (Immuno chemiluminescence assay, ICMA).

**Statistical analysis**

The results were expressed as mean±SEM. Data were analyzed by one-way analysis of variance. Sequential differences among means were evaluated at the level of p<0.05, using LSD contrast analysis as needed (SPSS version 20.0).

## Results

**Figure 1:**
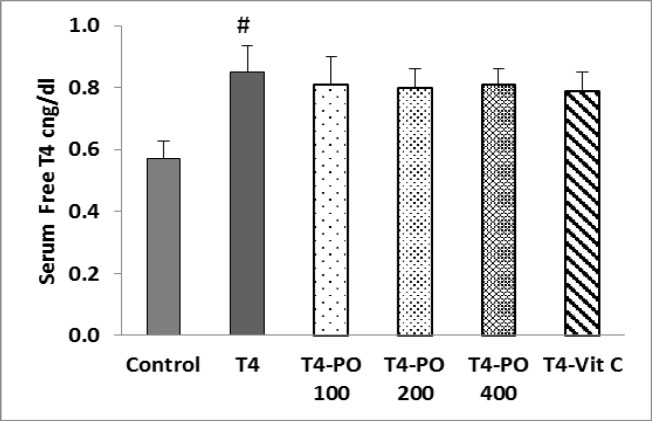
Serum free T4 level in different groups. Bars express mean±SEM. # p<0.05 compared to control group. T4-Po: T4 (20 μg/kg) plus 100, 200 and 400 mg/kg of *Portulaca oleracea* seeds extract, T4-Vit C: T4 (20 μg/kg) plus 100 mg/kg of vitamin C (n=8).

Serum free T4 level was significantly increased in T4 group compared to control group (p<0.05). This parameter was insignificantly increased in other treated groups (Figure 1).

**Figure 2:**
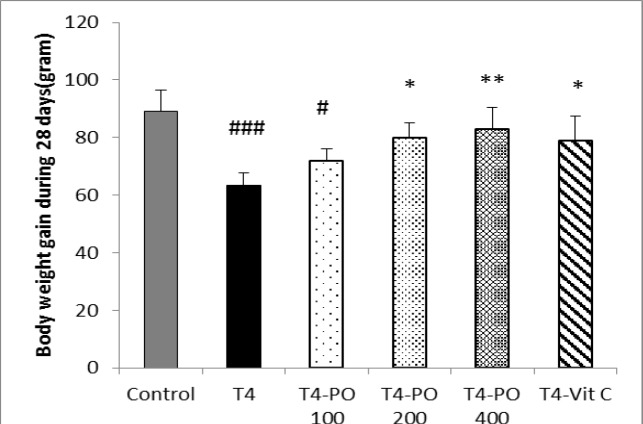
Body weight gain in different groups. Bars express mean±SEM (n=8). * p<0.05 and ** p<0.01 compared to T4 group. # p<0.05 and ### p<0.001 compared to control group. T4-Po: T4 (20 μg/kg) plus 100, 200, and 400 mg/kg of *Portulaca oleracea* seeds extract, T4-Vit C: T4 (20 μg/kg) plus 100 mg/kg of vitamin C.

Body weights were significantly decreased in T4 and T4-Po 100 groups compared to control group (p<0.001 and p<0.05, respectively). Administration of *P. oleracea*  200 and 400 mg/kg and vitamin C induced a significant increase in body weight when compared to T4 group (p<0.05, p<0.01 and p<0.05, respectively) (Figure 2).

**Figure 3:**
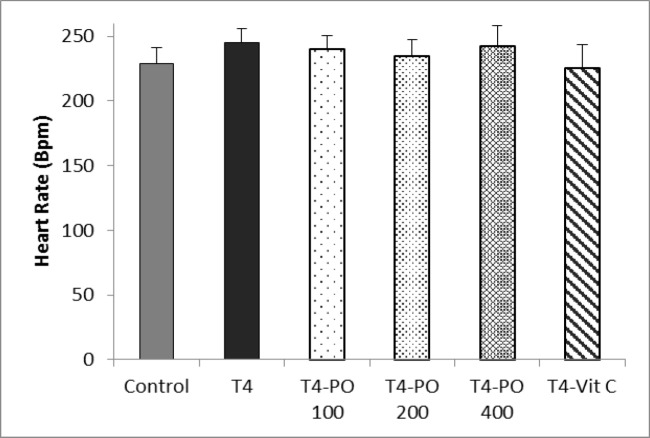
Heart rate in different groups. Bars represent mean±SEM. T4-Po: T4 (20 μg/kg) plus 100, 200, and 400 mg/kg of *Portulaca oleracea* seeds extract, T4-Vit C: T4 (20 μg/kg) plus 100 mg/kg of vitamin C (n=8).

Heart rate was insignificantly increased in all groups treated with levothyroxine sodium compared to control group (Figure 3).

**Figure 4:**
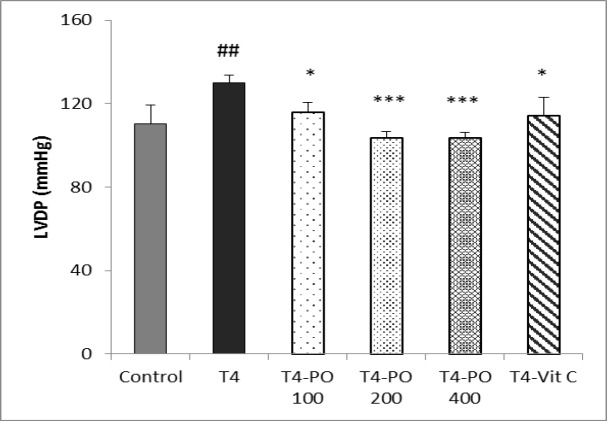
The mean of LVDP in different groups. Bars represent mean±SEM. * p<0.05 and *** p<0.001 compared to T4 group and ## p<0.01 compared to control group. LVDP: left ventricular developed pressure. T4-Po: T4 (20 μg/kg) plus 100, 200, and 400 mg/kg of *Portulaca oleracea* seeds extract, T4-Vit C: T4 (20 μg/kg) plus 100 mg/kg of

LVDP was significantly increased in T4 group compared to control (p<0.01). This parameter was decreased in T4-Po 100, 200, and 400 groups (p<0.05, p<0.001 and p<0.001, respectively) and T4-Vit C group (p<0.05) (Figure 4).

**Figure 5:**
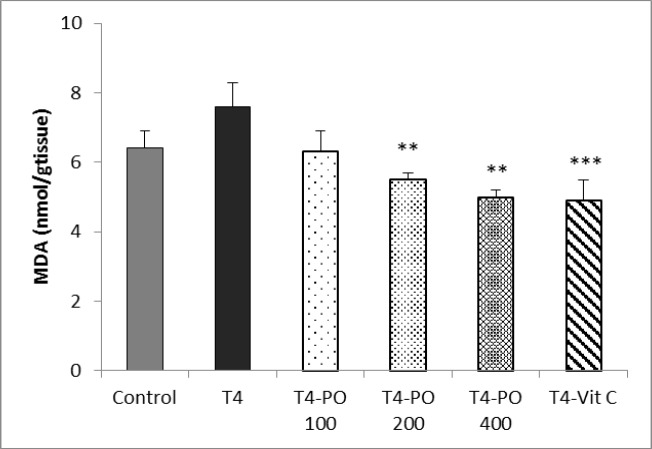
The level of MDA in different groups. Bars represent mean±SEM (n=8). **p<0.01 and *** p<0.001 compared to T4 group. T4-Po: T4 (20 μg/kg) plus 100, 200, and 400 mg/kg of *Portulaca oleracea* seeds extract, T4-Vit C: T4 (20 μg/kg) plus 100 mg/kg of vitamin C.

The level of MDA in heart tissue in T4 group was insignificantly increased compared to control group. Administration of *P*.* oleracea* extract at the doses of 200 and 400 mg/kg significantly reduced MDA level (both p<0.01) and vitamin C also significantly reduced MDA levels compared to T4 group (p<0.001) (Figure 5).

**Figure 6:**
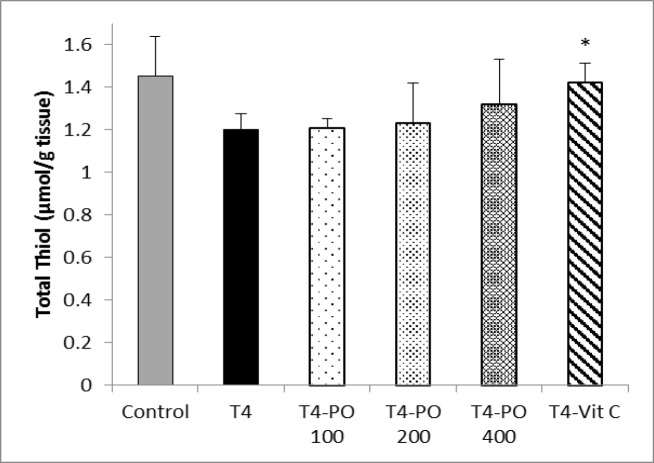
The level of total thiol groups in different groups. Bars represent mean±SEM (n=8). * p<0.05 compared to T4group.. T4-Po: T4 (20 μg/kg) plus 100, 200, and 400 mg/kg of *Portulaca oleracea* seeds extract, T4-Vit C: T4 (20 μg/kg) plus 100 mg/kg of vitamin C.

The level of total thiol in heart tissue was insignificantly decreased in T4 group compared to control group. Treatment with *P*.* oleracea* extract (200 and 400 mg/kg) insignificantly increased total thiol groups . There was no significant difference between T4-Po 100 and T4 groups. Administration of vitamin C significantly increased total thiol groups in comparison to T4 group (p<0.05) (Figure 6).

## Discussion

Subclinical hyperthyroidism induces mild symptoms and signs of thyrotoxicosis such as increased heart rate, atrial arrhythmias, increased left ventricular mass, impaired ventricular relaxation, increased systolic pressure, reduced exercise performance, and increased risk of cardiovascular death. All abnormalities are reversed by restoration of euthyroidism (Biondi et al., 2002 ▶).

In this study, administration of levothyroxine sodium at the dose of 20 µg/kg/day, increased free T4 level in all groups; however, neither *P. oleracea* extract nor vitamin C reduced serum levels free T4. These findings are consistent with a previous study which showed that injection of levothyroxine sodium (20 μg/kg/day) significantly increased T3 and T4 levels in rats (Wu et al., 2011 ▶). In another study, injection of three doses of levothyroxine (5, 15 and 20 µg/kg/day) was used to induce hyperthyroidism in rats; in all three groups, free T4 and free T3 levels significantly increased 24 hours after injection when compared to control group (Yu et al., 2015 ▶). 

We observed a significant decrease in body weight in T4 group versus control group. It has been reported that weight loss is one of the indicators of overt hyperthyroidism; but, in subclinical hyperthyroidism, there is no reduction in body weight although weight gain may be less than normal (Toft 2001 ▶).

Another study indicated that euthyroide state after treatment with T4 in hypothyroid people was associated with reductions in body weight but fat mass was unchanged and weight loss was primarily due to excretion of excess body water (Karmisholt et al., 2011 ▶). It has been shown that hyperthyroid patients have increased intake of carbohydrates, which reverses after treatment of the hyperthyroidism (Pijl et al., 2001 ▶). This may be considered as a factor involved in the body weight change. 

In the present study, it was shown that administration of *P. oleracea* at doses of 200 and 400 mg/kg and vitamin C caused a significant increase in body weight. These findings are consistent with a previous study which demonstrated that rats treated with lead acetate showed a significant decrease in body weight and maximum dose of vitamin C (1000 mg/kg) improved body weight (Mamoun et al., 2015 ▶).

In this study, the animals weight increased in *P. oleracea*-treated groups and this may be due to the temper-cooling properties and appetite-increasing activities of *P. oleracea* extract (Aghili 2009 ▶). In the present study, it was shown that blood pressure was increased in subclinical hyperthyroid group and *P. oleracea* extract reduced blood presure in a dose-dependent manner. It was indicated that although thyroid hormones reduce blood vessels resistance through production of NO, but total volume of blood is increased by renin-angiotensin aldosterone system activation and increased sodium reabsorption from the renal tubules, leading to further increase in blood volume, cardiac output and blood pressure (Okafor and Ezejindu 2014 ▶).*P. oleracea* seeds can reduce blood pressure which may due to its diuretic effects (Okafor and Ezejindu 2014 ▶). *P. oleracea* extract also has relaxant effect on skeletal (Okwuasaba et al., 1987 ▶) and smooth (Parry et al., 1988) muscles so it can reduce vascular resistance and decrease diastolic and systolic blood pressure.

The current study demonstrated that although heart rate was increased in T4 group and decreased in treated groups but none of them were significant. It has been reported that treatment with T4, increases heart rate in rats with subclinical hyperthyroidism (Gao et al. 2015 ▶). Our findings also showed that LVDP was significantly increased in subclinical hyperthyroidism group and this parameter was decreased in all *P. oleracea* -treated and vitamin C-treated groups. Various studies have indicated that increased levels of thyroid hormones, increase heart rate and LVDP (Chen et al. 2013 ▶; Marriott and McNeill 1983 ▶); these studies showed that heart weight increased about 15% following either T3 or T4 treatment while the increases in (+) or (-) dP/dt and LVDP were about 20-30%.

Lipids and proteins oxidation index was insignificantly increased in T4 group. Free radicals formation is one of the underlying mechanism(s) by excess production of thyroid hormones induced in subclinical hyperthyroidism; the increased lipid peroxidation in the liver and heart (Venditti et al. 1997 ▶) which were also observed in the current study may also be due to high level of thyroid hormones.

Li and colleagues also indicated that MDA concentration in the hippocampus of lead-poisoned rats treated with vitamin C was significantly lower than that in lead-poisoned control group (Li et al. 2008 ▶). Antioxidants have protective effect against myocytes toxicity induced by thyroid hormones. Constituents of *P. oleracea* seeds such as flavonoids (quercetin), omega-3, ascorbic acid, β- carotene and glutathione have antioxidant activities (Karimi et al. 2010 ▶); so, administration of this plant inhibits lipid peroxidation (Lim and Quah 2007 ▶) by scavenging free radicals and increasing intracellular concentration of glutathione. *P. oleracea* also has oleracein A, oleracein B and oleracein E; these phenolic alkaloids poses antioxidant activities (Sun et al., 2016 ▶). 

In another study, antioxidant activities of three phenolic alkaloids (oleracein A, oleracein B and oleracein E) isolated from *P. oleracea* were determined. The antioxidant activities of these phenolic alkaloids were lower than that of caffeic acid but higher than that of ascorbic acid and α-tocopherol. Oleracein E was the most potent compound that prevented MDA formation (Yang et al., 2009 ▶). Caffeic acid is a chemical found in many plants and foods. Coffee is the primary source of caffeic acid in the human diet. However, it can be found in other food sources such as apples, artichoke, berries, and pears. Caffeic acid is an antioxidant that also can inhibit carcinogenesis (Liao et al. 2003 ▶). The antioxidant effects of *P. oleracea* extract that were observed in this study in the heart tissue of rats with subclinical hyperthyroidism, were comparable with those reported previously.

According to the present results, *P. oleracea* extract has dose-dependent antioxidant properties. *P. oleracea* extract reduced blood pressure in a dose-dependent manner but had no beneficial effect on tachycardia. These results demonstrated that *P. oleracea* hydro-alcoholic extract reduces blood pressure and tissue level of MDA but increases weights in rats with subclinical hyperthyroidism; however, different doses of *P. oleracea* extract did not decrease free T4 level. The comparison of vitamin C and *P. oleracea* extract showed that *P. oleracea* extract was more effective in reducing blood pressure and elevation of weight gain while in terms of heart rate reduction, vitamin C was more effective.
